# Editorial: Songs and Signs: Interdisciplinary Perspectives on Cultural Transmission and Inheritance in Human and Nonhuman Animals

**DOI:** 10.3389/fpsyg.2022.953813

**Published:** 2022-06-17

**Authors:** Julia Hyland Bruno, Brian Boyd, David Rothenberg

**Affiliations:** ^1^Center for Science and Society, Columbia University, New York, NY, United States; ^2^Department of Anthropology, Columbia University, New York, NY, United States; ^3^Department of Humanities and Social Sciences, New Jersey Institute of Technology, Newark, NJ, United States

**Keywords:** cultural transmission, interdisciplinary/multidisciplinary, animal song, animal culture, non-anthropocentrism, learning biases, musicality, social learning

Songs and signs are learned beginning in infancy, persist and change across generations in seemingly all human societies, and may also characterize the lives of certain non-human animals, viz. the vocal traditions of songbirds and whales. Defined as shared behavior transmitted via social learning, culture is increasingly observed across disparate animal groups (Whiten, 2021), and scholarly research on animal cultures and how they change or endure is a primary focus of contemporary posthuman and non-anthropocentric approaches to the non-human animal (e.g., Mangano and Marrone, 2018). In the sciences, iterated-learning transmission chain experiments reveal that learning biases play an important role in shaping and stabilizing such diverse cultural behaviors as language and birdsong (Kirby et al., 2008; Fehér et al., 2009). Yet these convergent developments in different disciplines normally do not interface, and much remains unknown as to how social learning and genetic inheritance interact, or how different socially-inherited ways of living may be vulnerable or robust to anthropogenic changes transforming the planet.

We—an ethologist, an archaeologist/musician, and a musician/philosopher—created this Research Topic as a means of continuing the spirited conversation started at an interdisciplinary two-day conference held at Columbia University in February 2019, “The Transmission of Songs in Birds, Humans, and Other Animals,” which brought together diverse perspectives on how learned vocal traditions and other communicative systems are shaped by the ways in which they are learned. The present article collection, with less than half of the contributions authored by participants in the original conference, is the result of the efforts of over 80 referees and contributing authors hailing from traditional disciplines including psychology, biology, linguistics, anthropology, philosophy, and music, as well as inherently interdisciplinary fields: neuroscience, music cognition, music education, zoomusicology, sound studies, archaeoacoustics, and bioacoustics. Wherever possible, the articles in this collection were reviewed by at least one referee from a discipline other than the authors'. We were fortunate that many contributing authors were also willing to serve as referees, and for the extraordinary openness and good will we encountered throughout the project. Our hope with this collection is that the new space it opens up will enrich discipline-based as well as interdisciplinary work on animal cultures, and support nuanced thinking around conservation (Brakes et al., 2019).

We have organized the nineteen articles in the collection thematically into five sections, interweaving contributions from the lab and the wild: “Signal properties,” “The learner's role,” “Relational considerations,” “Sonic ecologies,” and “Modes of cultural transmission.” Each section represents multiple disciplines and an array of species and signal types: songbirds (over 25 species) appear in four sections (articles by Roeske et al.; Doolittle; Bilger et al.; Chopoorian et al.; Taylor; Williams; Lewis et al.; Garland and McGregor), as do marine mammals—dolphins (Meyer et al.) and humpback whales (Pénitot et al.; Mercado; Garland and McGregor; Sinclair et al.); two sections feature whistles (Meyer et al.; Verhoef and Ravignani). Finally, running through the collection (in particular articulated by Meyer et al.; Roeske et al.; Doolittle; Taylor; Pénitot et al.; Mercado; Rinsema and Edwards) is the cross-cutting theme of tuning our human senses to search for new ways of listening—to better hear other species, no less our own.

## Signal Properties

How do songs and signs become traditions? Although some cultural forms may be arbitrary [e.g., famously, words that lack iconicity (Saussure, 2011)], others may be conducive to cultural transmission. This section focuses on transmissibility and other properties of culturally-inherited signals. Meyer et al. introduce a comparative research agenda that links human whistled speech, used for long-distance communication in certain rural populations, and the long-distance, underwater whistle communication of dolphins. Whistled speech is language-specific and consists of a highly reduced version of the native language's voiced speech which nevertheless retains high intelligibility. Meyer et al. argue that studying how fluent listeners decode complex information from the degraded signals of whistled speech can help generate novel, testable hypotheses regarding the structure and function of dolphin whistles, which might turn out to be more complex than has been previously assumed. As whistles are adaptive in certain environments, their transmissibility in space is clearly related to their transmissibility in time (i.e., across generations). Socio-environmental pressures on cultural transmission are further explored in later sections of the article collection (“Relational considerations” and “Sonic ecologies”). The other three papers in this section deal with transmissibility on aesthetic grounds, following Darwin (1871), in terms of musicality. Both the universality of music and the possibility of non-human animal musicality are contested questions which have attracted attention across a number of fields in recent years (for reviews of these debates, see Verhoef and Ravignani; Taylor). Roeske et al., Doolittle, and Bilger et al. approach the musicality of birdsongs in inventive and complementary ways. Roeske et al. and Bilger et al. are multidisciplinary teams composed of musicians and scientists; Doolittle, a composer, integrates musical and ornithological perspectives in the interdisciplinary practice of zoomusicology, which she demonstrates by evaluating the songs of the hermit thrush according to nine areas in which music and birdsong can be said to overlap. The article by Roeske et al. is an exercise in perspective-taking as well: the authors deploy intuitions informed by expert listening to guide a quantitative analysis of mockingbird song, which yields the discovery of “compositional” rules at play in this species. Bilger et al. put the question of avian musicality directly to human listeners and find, intriguingly, that people perceive birdsongs from a range of species to be more musical than scrambled versions of the same sounds.

## The Learner's Role

Why should human musical appreciation and avian aesthetic preferences converge? Aesthetics can be an important driver of sexually-selected traits (Prum, 2017); they may also play a role in cultural transmission, through the intrinsic preferences (presumably shaped, at least in part, by genetic predispositions) of learners—who, after all, receive and transmit all of the songs and signs of culture. Human civilization would look very different if all cultural transmission proceeded like the game of Telephone, where copying error fuels the fun. However, transmission chain experiments show that even when signals are serially received and transmitted by learners in a Telephone-like setting, mutations on aggregate are not random, but rather expose underlying “universals” shared across individuals, resulting in biased iterated learning that constrains the space of cultural evolution (Bartlett, 1932; Kirby et al., 2008). In their study, Verhoef and Ravignani find that sets of slide whistle gestures transmitted by chains of experimental participants evolved to exhibit properties consistent with a set of putative melodic universals (Savage et al., 2015). Chopoorian et al., on the other hand, use an agent-based modeling approach, and probe how intrinsic learner bias may be indirectly affected by direct selective pressure on cultural traits. The results of their simulations demonstrate that learner preference cannot necessarily be inferred from the cultural landscape: whereas selection for rare cultural phenotypes led to a learning bias for novelty, selection for cultural homogeneity did not yield a corresponding conformity bias (see Williams for additional theoretical background on these and other mechanisms of cultural evolution). The experimental and theoretical contributions of Pfordresher et al. and Tichko et al., respectively, reaffirm that human cultural transmission is necessarily embodied. Our bodies constrain cultural behaviors differently toward different ends and at different times across the lifespan: Pfordresher et al. report cautionary findings that individual spontaneous production rates in speech, piano-playing, and tapping are consistent within but not necessarily correlated across modalities, while Tichko et al. present a dynamical theoretical framework of the ontogeny of musical rhythm, a universal enculturation process whose mechanisms remain largely mysterious.

## Relational Considerations

The previous section highlights various ways in which learners influence cultural transmission via non-random copying errors. The final section of the collection, “Modes of cultural transmission,” considers other, more agentic sorts of nonrandom change such as improvisation or innovation. In the present section, the authors assume learner agency, and focus on cultural transmission as a social process. Mueller, Stadler Elmer, and Taylor each take a close look at (listen to) the interaction dynamics of song teaching and learning—whether occurring in the context of popular media, formal didactics, or observed in a non-human animal—while Pénitot et al. describe an interspecies, musique concrète-inspired approach to learning about humpback whale song via an interactive human-whale interface loaded with bassoon sounds (chosen because of physical similarities between the bassoon and the humpback vocal apparatus). Mueller's history of late eighteenth-century German songs for families, which reveals how parents and children widely participated in a philanthropinist-orchestrated “knitting-together” of the family by learning scripted songs and performing them together, provides a clear example of how songs encode social relationships. Her article reminds us that while singing may have fundamental importance in human development (Trehub and Trainor, 1998), the songs sung by caregivers and infants are inescapably historical.

## Sonic Ecologies

This section extends the relational considerations of the previous section to include the full ecological context that songs inhabit and which may shape their transmission. Mercado's article on song morphing in humpback whales challenges the consensus view that humpback whale songs change as a result of cultural transmission (cf. Garland and McGregor). Cetaceans are one of only a few phylogenetic groups that exhibit vocal production learning (Petkov and Jarvis, 2012; Janik and Knörnschild, 2021). However, unlike in songbirds, there is so far no direct evidence that humpback whales acquire or alter their songs via social learning. As Mercado shows, songs appear to change in similar ways across years and geographically isolated populations, supporting a more parsimonious explanation of song change based on local interactions and/or other environmental conditions. Mercado concludes by breaking down the alternative predictions of the song copying vs. morphing hypotheses, with suggestions for obtaining more definitive evidence. Boren and Rinsema and Edwards also engage with the materiality of sound, exploring, respectively, how the acoustics of new liturgical spaces accompanied and even aided the Protestant Reformation, and how musical meaning and affordances for agency (e.g., in transformative pedagogies) are created by the migration of sonic materials from everyday sounds to song and back.

## Modes of Cultural Transmission

Just as human and non-human animal cultures vary, so too do modes of cultural transmission. The papers in this final section individually and collectively consider important sources of diversity in cultural evolution and underlying mechanisms. Species differences (Garland and McGregor; Sinclair et al.) are not the only differences considered: as Williams describes, synthesizing longitudinal studies of wild birds, different segments of the Savannah sparrow's song changed independently over the span of decades, consistent with the action of selection pressure from at least four different cultural evolutionary mechanisms. Lewis et al. also report a partitioning of variability in the song inheritance pattern of Java sparrows [a close relative of the zebra finch, a popular model organism, overrepresented in neuroethological studies of birdsong (Hauber et al., 2021)], which appear to exhibit high copying fidelity but performance differences related to their early developmental environment. Finally, Garland and McGregor and Sinclair et al. both take a comparative approach, with contributions that disentangle the factors governing, respectively, rates of cultural change, and the emergence of cumulative cultural evolution, a hallmark of human culture (Tomasello, 1999)—though, perhaps, neither uniformly important in the human cultural landscape, nor uniquely human as an achievement.

## Author Contributions

JHB, BB, and DR jointly wrote and revised the manuscript. All authors contributed to the article and approved the submitted version.

## Funding

JHB gratefully acknowledges the support of the Presidential Scholars in Society and Neuroscience program at Columbia University.

## Conflict of Interest

The authors declare that the research was conducted in the absence of any commercial or financial relationships that could be construed as a potential conflict of interest.

## Publisher's Note

All claims expressed in this article are solely those of the authors and do not necessarily represent those of their affiliated organizations, or those of the publisher, the editors and the reviewers. Any product that may be evaluated in this article, or claim that may be made by its manufacturer, is not guaranteed or endorsed by the publisher.
